# Bunyaviruses and the Type I Interferon System

**DOI:** 10.3390/v1031003

**Published:** 2009-11-23

**Authors:** Richard M. Elliott, Friedemann Weber

**Affiliations:** 1 Centre for Biomolecular Sciences, School of Biology, University of St. Andrews, St. Andrews, KY16 9ST, Scotland, UK; E-Mail: rme1@st-andrews.ac.uk; 2 Department of Virology, University of Freiburg, D-79008 Freiburg, Germany

**Keywords:** bunyaviruses, interferon system, NSs proteins

## Abstract

The family *Bunyaviridae* contains more than 350 viruses that are distributed throughout the world. Most members of the family are transmitted by arthopods, and several cause disease in man, domesticated animals and crop plants. Despite being recognized as an emerging threat, details of the virulence mechanisms employed by bunyaviruses are scant. In this article we summarise the information currently available on how these viruses are able to establish infection when confronted with a powerful antiviral interferon system.

## 1. Introduction

Viruses in the family *Bunyaviridae* are classified into five genera: *Orthobunyavirus, Phlebovirus, Hantavirus, Nairovirus, and Tospovirus* on the basis of molecular and serological characteristics. The term ‘bunyavirus’ refers to a member of the *Bunyaviridae* family, while the terms ‘orthobunyavirus’, ‘phlebovirus’, *etc*., refer to viruses in the eponymous genus. Members of the first four genera above are able to infect mammalian hosts, whereas the tospoviruses (which are not discussed here) are important plant pathogens. The majority of bunyaviruses is transmitted by arthropod vectors, such as midges, mosquitoes, sandflies, and ticks, and is able to replicate alternatively in these hosts. Generally there is greater specificity for the arthropod host than the vertebrate host, but some viruses, notably Rift Valley fever phlebovirus, are more promiscuous in that many different arthropod species and types of arthropod are competent vectors. Hantaviruses are the exceptions in not having an arthropod vector; instead these viruses are maintained in nature as persistent infections of rodents and humans become infected by inhaling aerosolised rodent excreta [1,2].

## 2. Diseases caused by the *Bunyaviridae*

Bunyaviruses cause four types of disease syndrome in man: fever, encephalitis, hemorrhagic fever and an acute respiratory illness (Table 1). A number of bunyaviruses are associated with self-limiting febrile illness, which although nonfatal, can be economically significant due to man-hours lost from work. Since many of these are found alongside malaria cases, diagnosis is often not achieved so the real extent of bunyavirus fevers is not known. Oropouche fever, which is the second most prevalent arboviral disease after Dengue fever in Brazil [3], is caused by Oropouche virus (OROV). Over the past 40 years recurrent epidemics, involving tens of thousands of patients, of this acute febrile illness have occurred in tropical areas of Central and South America [4]. A number of phleboviruses also cause febrile disease. Naples and Sicilian sandfly fevers are transmitted by *Phlebotomus* (sandfly) species in the Mediterranean basin [5], and are rapid onset, nonfatal illnesses with nonspecific symptoms (headache, photophobia, myalgia, *etc*.). Toscana virus (TOSV) causes aseptic meningitis in central Italy, and there is serological evidence for spread to neighbouring countries [6,7]. Punta Toro virus (PTV) has been repeatedly isolated from humans in Panama and Columbia where it causes acute febrile illness of short duration [8].

La Crosse virus (LACV) causes severe encephalitis and aseptic meningitis in children and young adults in the Midwestern United States [9]. Around 75 to 100 cases of La Crosse encephalitis requiring hospitalization are reported annually [10,11], and more than half require admission to intensive care units. In severe cases, long-lasting neurological sequelae are observed [9], with severe economic and social consequences [12]. Most LACV infections are clinically inapparent, though it is estimated that more than 300,000 infections occur annually in the Midwestern United States alone [10,11]. More recently, LACV infections have been reported in mid-Atlantic and southeastern regions, suggesting its distribution is wider than originally thought. Related orthobunyaviruses including California encephalitis, Jamestown Canyon, and snowshoe hare, have also been associated with human disease in North America, while Inkoo and Tahyna viruses cause influenza-like illness in Europe.

Viruses in all four genera that infect humans can cause hemorrhagic fevers. Rift Valley fever virus (RVFV) is a serious emerging pathogen affecting humans and livestock primarily in sub-Saharan Africa. Since the first description of an outbreak in Kenya in 1931 [13] there have been recurrent epidemics, killing thousands of animals, hundreds of humans, and causing significant economic losses [14]. RVFV has spread to Egypt (1977), and notably out of Africa to Yemen and Saudi Arabia (2000). RVFV is primarily a mosquito-transmitted disease of cattle, sheep and other ruminants, causing necrotic hepatitis, hemorrhage and abortion [15,16]. Humans are usually infected by close contact with infected animal material, and disease symptoms including temporarily incapacitating febrile illness, retinitis, meningoencephalitis, and, in about 1% of cases, hemorrhagic fever [14]. The apparent ease with which RVFV can spread to new geographical areas, coupled with its capability to cause major epidemics in livestock and humans have prompted authorities to list RVFV as a notifiable disease and a potential biological weapon [17].

The orthobunyavirus Ngari (NRIV; originally designated Garissa virus), was isolated from hemorrhagic fever cases during investigation of a RVFV outbreak in East Africa in 1997 and 1998 [18]. Molecular characterisation of the virus indicated that two genome segments were almost identical to those of the prototypic bunyavirus Bunyamwera virus (BUNV; a relatively benign human pathogen) while the third segment was similar to that of Batai virus (BATV; [19–21]). Thus NRIV is likely to have arisen by genome segment reassortment, illustrating the potential of reassortment to generate viruses with enhanced pathogenicity.

Crimean-Congo hemorrhagic fever virus (CCHFV) is a tick-borne nairovirus causing severe infections in Africa, Asia and Eastern Europe [22,23]. CCHFV was originally described in the 1940s during an outbreak of hemorraghic fever in the Crimea, with a case fatality rate of 15–30%. Subsequently, it was shown to be antigenically identical to a virus isolated from a febrile patient in the Congo. Many non-human mammalian species and also ostriches can be infected by CCHFV though there is no apparent disease. Human infections are acquired by tick bite, contact with infected animal blood or nosocomially when treating infected patients.

Hantaviruses are associated with two major disease syndromes in man – hemorrhagic fever with renal syndrome (HFRS) and hantavirus cardio-pulmonary syndrome (HPS). Clinically, HFRS has long been recognized, and ranges in severity from a mild illness, nephropathia epidemica, mostly prevalent in Fenno-Scandinavia, to the severe form in the Eastern Europe and Asia. In China there are about 200,000 hospitalised cases per annum of HFRS caused by Hantaan (HTNV) and Seoul (SEOV) viruses, with mortality reaching 5–15%. In 1993, a sudden outbreak of an acute respiratory disease led to the isolation of a new hantavirus, now called Sin Nombre virus (SNV), in the United States [24]. Subsequently, many more new hantaviruses were discovered throughout the Americas, a number of which cause HPS. The overall case fatality of HPS is around 40%.

## 3. Virus particles and infection cycle

Bunyavirus particles are about 100 nm in diameter, and comprise a lipid envelope containing two viral glycoproteins (termed Gn and Gc) that contains the tripartite RNA genome in the form of ribonucleoprotein (RNP) complexes. By negative-staining bunyavirus particles appear pleomorphic in the electron microscope[1], but recent structural analyses of the phleboviruses Uukuniemi virus (UUKV) and RVFV by cryoelectron microscopy revealed a surprising level of order with a spherical shape and Gn/Gc capsomers organized on a T=12 icosahedral lattice [25–28].

**Figure 1.**
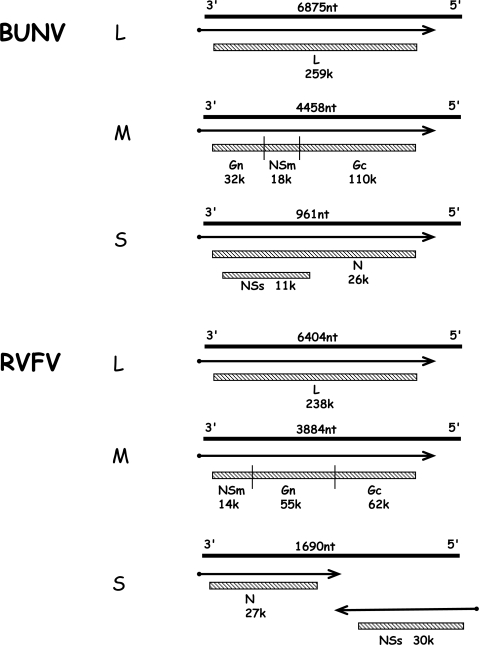
BUNV orthobunyavirus and RVFV phlebovirus coding strategies (not to scale). The three genomic RNA segments L, M and S are shown as solid lines with their lengths (nt) shown above. mRNAs are indicated by arrows, with solid dot depicting nontemplated primer at 5′end. Gene products are shown as hatched boxes, and protein designations and sizes (kDa) are indicated. The ambisense S segment of RVFV encodes proteins in both negative- and positive-sense orientations, separated by an intergenic region that can form a hairpin structure.

The three single-stranded RNA segments are of negative polarity and are encapsidated by the nucleocapsid (N) protein to form the RNP; associated with the RNPs are a few copies of the viral L protein, the RNA dependent RNA polymerase. All bunyaviruses encode four structural proteins: the viral polymerase (L) on the large (L) segment, the glycoproteins (Gn and Gc) on the medium (M) segment, and the N protein on the small (S) segment. Viruses within the *Bunyavirus* and *Phlebovirus* genera also encode non-structural proteins, either on the M segment (termed NSm) and/or on the S segment (NSs). The phlebovirus NSs protein is encoded in an ambisense strategy. Figure 1 shows the genomic organisation of BUNV (genus *Orthobunyavirus*), and of RVFV (genus *Phlebovirus*) that encode the maximal set of six viral proteins, *i.e.* L, Gn, Gc, NSm, N and NSs [1].

Bunyavirus replication occurs in the cytoplasm and virion assembly and budding take place at membranes of the Golgi apparatus. The first step in the infectious cycle after virus entry is primary transcription of the genomic negative-sense RNA by the virion-associated RNA polymerase. Viral mRNA synthesis is primed by 5′ end sequences of cellular mRNAs that are cleaved by an endonuclease activity in the L protein. Hence, bunyavirus mRNAs are capped and contain short heterogeneous oligonucleotides, 12 to 18 nt in length, at their 5′ ends. Viral mRNA transcription terminates at a signal 50 to 150 nt before the end of the genomic template RNA [29–31], but the mRNAs are not demonstrably polyadenylated. After translation of viral proteins, RNA synthesis switches to replication mode which involves the primer-independent synthesis of a full-length, exact copy complementary positive-sense RNA (called the the antigenome) that acts as template for the synthesis of progeny negative-sense genomes. Both the genome and antigenome RNAs are only found encapsidated by N protein as RNPs, whereas mRNAs are naked to allow ribosomal access.

## 4. Innate immune responses – the type I interferon system

Type I interferons (IFN-α/β) are produced and secreted by cells in response to virus infection. They activate in neighboring cells the expression of so-called IFN-stimulated genes (ISGs). Many ISGs encode proteins with the ability to directly or indirectly inhibit virus multiplication. Three phases of the cellular IFN response, namely induction, signaling, and effector mechanisms, can thus be distinguished.

IFN induction in infected fibroblasts occurs mainly by an intracellular pathway. Viral RNA products such as double-stranded (ds) and 5′-triphosphorylated single-stranded (ss) RNA trigger a signaling chain which activates IFN-β gene expression [32]. Two RNA helicases, RIG-I and MDA5 (collectively termed RIG-like receptors, RLRs), are the main intracellular receptors of viral RNA [33,34], but the dsRNA-binding protein kinase PKR can also contribute [35–37]. RIG-I binds to short dsRNA molecules whereas MDA5 activation is more dependent on long dsRNA structures [38]. Moreover, RIG-I has the ability to bind the triphosphate groups on the 5′-end of uncapped viral ssRNA [39–41]. Negative-strand RNA viruses do not produce substantial amounts of dsRNA during infection [42] but are strong activators of RIG-I [43]. Most likely, this is due to the triphosphate group at the 5′ end of their ssRNAs [39–44].

The binding of a viral RNA to RIG-I and MDA5 induces a signalling chain which eventually results in the phosphorylation of the transcription factor IRF-3 [45], a member of the IFN regulatory factor (IRF) family [46]. Phosphorylated IRF-3 homo-dimerizes and moves into the nucleus where it initiates IFN-β mRNA synthesis. In addition to IRF-3, the transcription factors IRF-7, NF-κB and AP-1 are triggered by viral replication to enhance IFN gene expression [47].

Besides the intracellular RLRs, the endosomally localized toll-like receptors TLR-3 and TLR-7/8 are capable of recognizing viral dsRNA and ssRNA, respectively [48,49]. Binding of the specific ligands to the TLRs results in activation of a signaling chain which induces IRF- and NF-κB-dependent IFN transcription.

All IFN-α/β subtypes bind to and activate a common type I IFN receptor which is present on virtually all host cells [50]. Binding of IFN-α/β activates the so-called JAK-STAT signalling pathway. The signal transducer and activator of transcription (STAT) proteins are latent cytoplasmic transcription factors which become phosphorylated by Janus kinase (JAK) family members [51]. Phosphorylated STAT-1 and STAT-2 recruit a third factor, IRF-9, and translocate to the nucleus to activate the promoters of ISGs.

There are more than 300 ISGs which have antiviral, antiproliferative, and immunomodulatory functions [52]. IFN-induced proteins include enzymes, transcription factors, cell surface glycoproteins, cytokines, chemokines and a large number of factors with unknown function. The three main proteins with direct antiviral activity are the Mx GTPases, the protein kinase R (PKR), and the 2′–5′ oligoadenylate synthetases (2–5 OAS)/RNaseL system. Mx proteins belong to the superfamily of dynamin-like large GTPases and have been discovered as mediators of genetic resistance against orthomyxoviruses in mice [53]. The human MxA protein blocks replication of the infecting virus soon after cell entry by targeting and mis-sorting viral RNPs [54–56]. PKR and 2–5 OAS are constitutively expressed in a latent, inactive form. Basal mRNA levels are upregulated by IFN-α/β and these enzymes need to be activated by viral dsRNA. PKR is also activated by ssRNA containing a 5′ triphosphate group and a short stem-loop [57] and by influenza virus nucleocapsids [58]. PKR is a serine-threonine kinase that phosphorylates the alpha subunit of the eukaryotic translation initiation factor eIF2 [59], thus blocking translation of cellular and viral mRNAs. The 2–5 OAS catalyses the synthesis of short 2′–5′ oligoadenylates [60] that activate the latent endoribonuclease RNaseL which in turn degrades both viral and cellular RNAs [61]. Apart from these, the RNA-specific adenosine deaminase 1 (ADAR 1), viperin, and the products of the ISG56 (p56) and ISG20 genes have also been shown to have antiviral activity [62,63].

Given the high efficiency of the IFN system to combat virus infections, pathogenic viruses had to evolve means to circumvent or inactivate the IFN system. So-called viral IFN antagonists interfere with IFN induction, IFN signaling, the action of particular IFN effector proteins, or a combination thereof [33,64].

## 5. IFN responses to bunyaviruses

An age-dependent susceptibility to many bunyavirus diseases exists for humans as well as for livestock and laboratory animals [15,65,66]. This may suggest that the initial host resistance is mediated by the type I IFN system, which is not fully matured in young animals [67,68]. Indeed, growth of several bunyaviruses can be inhibited by IFNs [69–75]. Moreover, mice lacking a functional type I IFN receptor are highly susceptible to infection with BUNV, LACV, Dugbe nairovirus (DUGV), HTNV, and RVFV [76–81].

Of the IFN-stimulated effector proteins which may mediate these effects, Mx proteins seem to be a main factor as they were shown to confer a strong protective effect against the orthobunyavirus LACV, the phlebovirus RVFV, the nairovirus CCHFV, and several hantaviruses [79,82–86]. Strikingly, introduction of the MxA gene into mosquito cells was sufficient to inhibit replication of LACV [87]. Mx proteins can inhibit primary transcription [84] as well as genome replication [83,88] of bunyaviruses. An interaction of human MxA with the viral N protein was demonstrated for LACV, BUNV, RVFV, and CCHFV [56,82,88], supporting the model that copolymers of Mx with N or RNPs affect viral polymerase function [55,56].

The IFN system still delays bunyavirus replication in systems lacking functional Mx genes [69,70,77,89,90]. In line with this, for the orthobunyavirus BUNV it was shown that PKR contributes to host resistance *in vivo*, whereas the 2–5 OAS/RNaseL system has no effect [91]. The phlebovirus RVFV is also sensitive to PKR *in vitro* and *in vivo*, but only in the absence of the viral NSs protein [70,92]. Interestingly, DUGV nairovirus is restricted by IFN but neither Mx nor PKR play a role *in vivo* [78], although some effect of MxA was measured in cell culture [93]. Collectively, these studies suggest that, besides Mx and PKR, additional anti-bunyaviral IFN effectors exist.

## 6. IFN induction by bunyaviruses

Several *in vivo* studies indicate that production of IFNs is an important determinant of bunyavirus pathogenesis. A late onset of the IFN response was correlated with increased susceptibility of rhesus monkeys to RVFV [94], and a mouse model of RVFV infection revealed very little IFN production [77]. For the related phlebovirus PTV it was shown that virulence in Syrian hamsters depends on the ability of virus strains to delay IFN induction [95,96]. Similarly, human pathogenic hantaviruses suppress IFN induction or signaling more efficiently than their non-pathogenic relatives [97–101], and in hantavirus-infected patients type IFN levels did not increase during the course of disease [102].

For RNA viruses, the main structures eliciting IFN induction are dsRNA and 5′ triphosphate-containing ssRNA [32,48,49]. Bunyavirus-infected cells do not contain significant amounts of dsRNA [42], most likely because packaging of genomes and antigenomes into RNPs minimizes basepairing. However, activation of TLR-3 (by PTV and HTNV) and of PKR (by BUNV and RVFV) could indicate the presence of low but biologically active levels [70,91,92,99,103]. PKR, however is activated by a host of other agents besides dsRNA [63], including 5′ triphosphate ssRNA [57]. How PKR is in fact activated by BUNV or RVFV has not been investigated so far. However, it is clear that the genome of orthobunyaviruses and phleboviruses does contain the 5′ triphosphate group ([44] and unpublished data). By contrast, the genomes of nairoviruses (represented by CCHFV) and hantaviruses (represented by HTNV) are monophosphorylated at their 5′ ends [44,104]. The genomes of RVFV and LACV are hence strong triggers of RIG-I-dependent IFN induction, whereas those of CCHFV and HTNV are not ([44] and unpublished data). Thus, bunyaviruses infecting animals can be distinguished into those whose genomes contain the RIG-I- and PKR-activating 5′ triphosphate group (orthobunya- and phlebo-viruses) and those where it is apparently removed during infection (nairo- and hanta-viruses).

In the case of nairo- and hantaviruses, therefore, it is conceivable that molecular patterns other than RNA are important for virus recognition. Recent results obtained for SNV suggest that cytokine induction can occur by virus particles alone and independent of IRF-3, IRF-7, RIG-I, MDA5, or TLR pathways [105,106]. On the other hand, however, several other studies did not observe significant innate immune activation by replication-defective hantavirus particles [99,101,107,108] suggesting strain- or protocol-dependent differences. Indications of non-canonical virus recognition have also been found for other enveloped viruses [109–111], and may be mediated by glycoprotein binding. Interestingly, for alphaviruses it was found that mosquito cell-derived virus preparations were poor IFN inducers in myeloid dendritic cells, whereas mammalian cell-derived viruses exhibited strong induction [112]. This striking difference is most probably caused by differences in glycosylation, cholesterol content, or RNA modifications. A similar scenario is possible for those bunyaviruses that use arthropods as vectors. Thus, depending on their genome 5′ ends and glycosylation patterns, bunyaviruses may activate cytokine production both by well-described and by non-canonical pathways.

## 7. Viral countermeasures

The segmented nature of the bunyavirus genome implies that orthobunyaviruses and phleboviruses carry three RIG-I-activating 5′ triphosphate-containing ssRNAs per infecting particle. To counterbalance this, these viruses express a highly active antagonist of IFN induction, the NSs protein [77,90,113]. Although being different in sequence, size, and mode of expression (see Figure 1), both the orthobunyavirus and the phlebovirus NSs proteins act by blocking host cell RNA polymerase II (RNAP II). The NSs protein of RVFV interacts with the p44 subunit of the essential transcription factor TFIIH; it forms filamentous structures with p44 in the nucleus that also contain the XPD subunit of TFIIH [114]. In this way NSs sequesters p44 and XPD, thus blocking assembly of TFIIH and resulting in a decrease in overall host transcription. Moreover, during the early phase of infection RVFV NSs specifically inhibits the IFN-β promoter by recruiting the repressor protein SAP30 [115]. BUNV NSs acts by interfering with phosphorylation of the carboxy terminal domain of the large subunit of RNAP II, which also results in a decrease in host transcription [116]. The BUNV NSs protein interacts with the MED8 component of Mediator, a protein complex necessary for mRNA production. The interacting domain on NSs was mapped to the C-terminal region, and a recombinant virus in which this domain was deleted had strongly reduced ability to inhibit host protein expression. In addition, the virus expressing the truncated NSs protein was unable to inhibit the interferon response, and behaved similarly to a virus lacking NSs entirely [117]. The NSs of the orthobunyavirus LACV acts in a similar manner by causing a general host cell shut-off [118] to abrogate the transcription of IFN genes [76]. Reassortment and overexpression studies suggest that the phlebovirus PTV also expresses an NSs protein blocking IFN induction [96], and using reverse genetics it was shown that NSs of SFSV can replace RVFV NSs as an inhibitor of IFN induction [70]. For LACV, PTV, and SFSV the mechanism and molecular target of NSs are not known so far. Thus, the function (though not the sequence) of NSs appears to be highly conserved across the orthobunya- and phlebovirus genera. However, recent nucleotide sequence analysis showed that naturally-occurring isolates from three orthobunyavirus serogroups (Anopheles A, Anopheles B, Tete) do not encode an NSs protein [119]. Most of these NSs-null viruses failed to prevent IFN induction except for Tacaiuma virus (TCMV), which behaved like wt BUNV. Strikingly, TCMV is the only one of those viruses known to cause human disease. In agreement with a previous study on LACV [76], these observations support the view that the NSs proteins expressed by orthobunyaviruses and phleboviruses have evolved to combat the IFN system of vertebrates. Moreover, TCMV appears to employ an alternative anti-IFN mechanism.

For hantaviruses and nairoviruses, the above-mentioned removal of the 5′ terminal triphosphate from the genomic ssRNA most likely represents a general strategy to avoid RIG-I dependent IFN induction [44]. Besides this, the L protein of CCHFV contains an ovarian tumor (OTU) domain allowing innate immune evasion [120]. This domain is also present on the L protein of the related nairoviruses DUGV and Nairobi sheep disease virus, but not of the phlebovirus RVFV. The OTU domain represents a superfamily of ubiquitin (Ub)-deconjugating proteases found in prokaryotes, eukaryotes, and viruses. Ub and IFN-stimulated gene product 15 (ISG15) are short proteins which are covalently conjugated to other proteins and mediate innate antiviral responses. Frias-Staheli *et al*. showed that the OTU domain-containing proteases from CCHFV and related nairoviruses deconjugate Ub and ISG15 from cellular target proteins [120]. Expression of the viral OTU domain antagonizes the antiviral effects of ISG15 and inhibits NF-κB-dependent signaling.

For hantaviruses, an additional +1 open reading frame (ORF) within the N ORF is conserved in Puumala virus (PUUV)-like and SNV-like viruses, but not in others [121]. Some evidence for the expression of an NSs protein has been obtained for PUUV and Tula hantavirus (TULV), but the protein only weakly suppressed IFN induction if over-expressed in cell culture [122]. In support of a biological function, it was shown that the presence of an intact NSs ORF conferred an IFN-dependent growth advantage for TULV [123]. The conservation of the NSs ORF across the hantavirus genus however does not correlate with virulence. The lack of a reverse genetics system for hantaviruses, however, is hampering further characterization of this gene product. Thus, additional, disease-connected mechanisms appear to be in place, as differences in cytokine profiles exist between pathogenic and non-pathogenic hantaviruses (see above). In line with this, for the pathogenic New York-1 hantavirus (NY-1V), but not for the apathogenic Prospect Hill hantavirus (PHV), it was found that the cytoplasmic tail domain of the Gn glycoprotein downregulates IFN induction by interacting with TRAF3, an important adaptor protein for IRF-3 and NF-κB signaling [97,124]. The C termini of Gn proteins of NY-1V as well as of ANDV and HTNV, but not of PHV, are subject to proteasomal degradation [125], again suggesting virulence-determining differences. Other pathogenic hantaviruses may employ different mechanisms, since the glycoprotein of the virulent ANDV (a weak IFN inducer) was unable to counteract IRF-3 activation [101]. In fact, the N protein of HTNV was shown to sequester NF-κB by interacting with importin-α [126], suggesting a further mode of inhibiting cytokine synthesis by hantaviruses.

Some bunyaviruses were shown to also interfere with IFN signaling. The hantaviruses ANDV, HTNV, NY-1V and PHV, as well as CCHFV and the orthobunyavirus OROV were insensitive to IFN if applied after establishment of infection [71,97,101,109]. For hantaviruses, this is most probably because the viral glycoproteins downregulate IFN-induced STAT-1/2 activation, as shown for ANDV and PHV [101]. For orthobunyaviruses like OROV, this may be due to the NSs protein, because the NSs-null TCMV failed to counteract IFN action independent of the time of application [71].

The antiviral action of IFN is inhibited by the OTU domain of CCHFV by interfering with ubiquitin- and ISG15-dependent signaling pathways [120]. A recombinant Sindbis virus carrying the OTU gene was able to overcome ISG15-mediated protection from lethal infection. Moreover, the NSs of RVFV was recently shown to promote the specific degradation of PKR through the proteasomal pathway [70,92]. Hence, RVFV mutants lacking NSs are more sensitive to the antiviral action of PKR than wt RVFV is. Moreover, wt mice can resist infection with an NSs-deleted RVFV mutant, whereas PKR knockout mice succumb to it [70]. Since these wt mice do not express a functional Mx gene, this supports the view that PKR is an important anti-bunyaviruses IFN effector besides Mx. RVFV escapes this by degrading PKR, but the NSs of SFSV and LACV were not capable of this, suggesting that the destruction of PKR contributes to the high virulence of RVFV [70].

## 8. Conclusions

Despite the significant economic and medical impact of this huge RNA virus family, we are only beginning to understand the interactions of bunyaviruses with the IFN system. Neither induction pathways nor viral IFN antagonists have been systematically characterized, let alone compared between the different family members. Such data are urgently needed, as virus-IFN system interactions are critical determinants of pathogenesis. Moreover, viruses with targeted deletions in their IFN-antagonistic functions are excellent candidates for live virus vaccines. They can be grown to high titers in IFN-deficient cell cultures [127] but are attenuated *in vivo* since they elicit robust innate and adaptive immune responses. This concept has been proven for influenza viruses [128–131], and several other viruses (reviewed in [132]), and was recently applied to RVFV with success [133]. Also, given the susceptibility of bunyaviruses to the antiviral effect of IFN, interfering with viral IFN antagonism or enhancing PRR recognition by pharmacological means could be a therapeutic possibility in the future. Thus, a better understanding of the interplay between bunyaviruses and the IFN response can help to design new strategies for prevention and therapy.

## Figures and Tables

**Table 1.** Selected bunyaviruses infecting humans and domestic animals.

**Genus/ virus**	**Disease**	**Vector**	**Distribution**
***Orthobunyavirus***			
La Crosse (LACV)	Human: encephalitis	Mosquito	N America
Ngari (NRIV)	Human: hemorrhagic fever	Mosquito	Africa
Oropouche (OROV)	Human: fever	Midge	S. America
Tahyna	Human: fever	Mosquito	Europe
***Phlebovirus***			
Punta Toro (PTV)	Human: fever	Sandfly	M America
Rift Valley fever (RVFV)	Human: encephalitis, hemorrhagic fever, retinitis, fatality 1%.	Mosquito	Africa
	Domestic ruminants: necrotic hepatitis, hemorrhage, abortion		
Sicilian sandfly fever (SFSV)	Human: fever	Sandfly	Europe, Africa
Toscana (TOSV)	Human: fever	Sandfly	Europe
***Hantavirus***			
Hantaan (HTNV)	Human: severe hemorrhagic fever with renal syndrome (HFRS), fatality 5–15%	Field mouse	Eastern Europe, Asia
Puumala (PUUV)	Human: mild HFRS, fatality 0.1%	Bank vole	Western Europe
Seoul (SEOV)	Human: moderate HFRS, fatality 1%	Rat	Worldwide
Sin Nombre (SNV)	Human: hantavirus cardiopulmonary syndrome, fatality 40%	Deer mouse	N America
***Nairovirus***			
Crimean-Congo hemorrhagic fever (CCHFV)	Human: hemorrhagic fever, fatality 20–80%	Tick, culicoid fly	Eastern Europe, Africa, Asia
Nairobi sheep disease	Sheep, goat: fever, hemorrhagic gastroenteritis, abortion	Tick, culicoid fly, mosquito	Africa, Asia
